# Facial nerve outcome score: a new score to predict long-term facial nerve function after vestibular schwannoma surgery

**DOI:** 10.3389/fonc.2023.1153662

**Published:** 2023-06-12

**Authors:** Giuseppe Di Perna, Raffaele De Marco, Bianca Maria Baldassarre, Enrico Lo Bue, Fabio Cofano, Pietro Zeppa, Luca Ceroni, Federica Penner, Antonio Melcarne, Diego Garbossa, Michele Maria Lanotte, Francesco Zenga

**Affiliations:** ^1^ Department of Neuroscience “Rita Levi Montalcini”, University of Turin, Turin, Italy; ^2^ Skull Base and Pituitary Surgery Unit, “Città della Salute e della Scienza” University Hospital, Turin, Italy; ^3^ Spine Surgery Unit, Casa di Cura "Città di Bra", Bra, Cuneo, Italy; ^4^ Spine Surgery Unit, Humanitas Gradenigo Hospital, Turin, Italy; ^5^ Department of Psychology, University of Turin, Turin, Italy; ^6^ Neurosurgery Unit, “Città della Salute e della Scienza” University Hospital, Turin, Italy; ^7^ Functional, Oncological and Stereotactic Neurosurgery Unit, “Città della Salute e delle Scienza” University Hospital, Turin, Italy

**Keywords:** vestibular schwannoma, restrosigmoid approach, facial nerve, facial nerve function, intraoperative neuromonitoring, outcome score

## Abstract

**Introduction:**

Patients’ quality of life (QoL), facial nerve (FN), and cochlear nerve (CN) (if conserved) functions should be pursued as final outcomes of vestibular schwannoma (VS) surgery. In regard to FN function, different morphologic and neurophysiological factors have been related to postoperative outcomes. The aim of the current retrospective study was to investigate the impact of these factors on the short- and long-term FN function after VS resection. The combination of preoperative and intraoperative factors resulted in designing and validating a multiparametric score to predict short- and long-term FN function.

**Methods:**

A single-center retrospective analysis was performed for patients harboring non-syndromic VS who underwent surgical resection in the period 2015–2020. A minimum follow-up period of 12 months was considered among the inclusion criteria. Morphological tumor characteristics, intraoperative neurophysiological parameters, and postoperative clinical factors, namely, House–Brackmann (HB) scale, were retrieved in the study. A statistical analysis was conducted to investigate any relationships with FN outcome and to assess the reliability of the score.

**Results:**

Seventy-two patients with solitary primary VS were treated in the period of the study. A total of 59.8% of patients showed an HB value < 3 in the immediate postoperative period (T1), reaching to 76.4% at the last follow-up evaluation. A multiparametric score, Facial Nerve Outcome Score (FNOS), was built. The totality of patients with FNOS grade A showed an HB value < 3 at 12 months, decreasing to 70% for those with FNOS grade B, whereas 100% of patients with FNOS grade C showed an HB value ≥ 3. The ordinal logistic regression showed three times increasing probability to see an HB value ≥ 3 at 3-month follow-up for each worsening point in FNOS score [Exp(B), 2,999; p < 0.001] that was even more probable [Exp(B), 5.486; p < 0.001] at 12 months.

**Conclusion:**

The FNOS score resulted to be a reliable score, showing high associations with FN function both at short- and long-term follow-up. Although multicenter studies would be able to increase its reproducibility, it could be used to predict the FN damage after surgery and the potential of restoring its function on the long-term period.

## 1 Introduction

Facial nerve (FN) function after surgery for vestibular schwannoma (VS) highly influences the quality of life (QoL) of the patients (1–7). More than gross total resection (GTR), preserving FN function is a primary concern along with hearing, if did not affect, during VS surgery. Although optimal percentages of anatomical preservation of the FN (reaching 95%) have been described, functional preservation rates are still lower, varying from 70 to 90% in different series (8, 9).

To preserve this function, surgical strategy has been moved from GTR to near-total (NTR) or subtotal resection (STR) balancing functional integrity preservation with disease’s control and respecting the concept of “maximal safe resection”, which is broadly diffused in skull base surgery (10, 11).

However, despite surgical strategy to preserve FN function (12) and the introduction of this “sparing surgery policy” (13), FN palsy still continues to represent the main source of morbidity related to VS surgery.

The possibility to predict long-term outcome of FN function could anticipate the patient that can benefit from an early procedure of facial-hypoglossal anastomosis (14).

Different factors resulted to be associated to postoperative FN function, independently or in association, but only few studies have proposed specific practical tools to anticipate FN function (15–18).

Therefore, the aim of this study is to evaluate the effect of morphological and neurophysiological factors on short- and long-term FN function. In addition, by combining factors significantly capable of influencing nerve function, the current study aims to build a new multi-parametric score defining its validity and association with short- and long-term FN function.

## 2 Materials and methods

This is a retrospective study analyzing prospectively collected data of patients undergoing surgery for VS removal by a team of experienced surgeons composed of neurosurgeons and otolaryngologists, at the authors’ institution from 2015 to 2020.

Adult patients (age ≥ 18 years) with primary diagnosis of non-syndromic vestibular schwannoma were included in the study. Conversely, patients with preoperative FN deficiency and patients undergoing facial-hypoglossal anastomosis were excluded from the study. Finally, unavailability of clinical, radiological, and intra- and postoperative data, and a minimum 12-month follow-up resulted in exclusion from the study. All inclusion and exclusion criteria were summarized in (**Supplementary Table 1**).

The following data were collected for the study: biographical data, age, sex, tumor type, tumor size, tumor morphology, preoperative neurological status, type of surgical approach, intraoperative FN stimulation data, extent of resection, complications, length of surgery, histological examination, and clinical data regarding FN function assessed both postoperatively and during follow-up.

### 2.1 Neuroimaging

Tumor size and its relationships with surrounding CPA (Cerebellopontine angle) structures were analyzed using T1-weighted (T1w) sequences with gadolinium and FIESTA (fast imaging employing steady-state acquisition) or CISS (constructive interference in steady state) nuclear magnetic resonance imaging (MRI) sequences. Specifically, tumor size was measured in millimeters, using the largest diameter of the extra-meatal portion of the tumor measured on axial cuts of T1w sequences with gadolinium, according to the international measurement criteria (19, 20). Tumors with diameters greater than 30 mm were classified as large tumors, whereas tumors with diameters less than 30 mm were classified as small tumors.

On the basis of the presence or absence of the cystic component, either central or peripheral, tumors were classified morphologically as cystic and non-cystic (21).

VS relationships with the internal acoustic meatus and CPA structures was assessed by using two of the most used classification systems, namely, the Koos and the Samii classifications (22–24).

### 2.2 Surgical technique and intraoperative neurophysiological monitoring

A retrosigmoid approach to the CPA was performed in all cases. Patient was positioned in lateral position with the head fixed to a Mayfield headrest, slightly flexed and rotated so that the mastoid tip resulted to be the highest point. A lumbar drain was placed in all patients to facilitate intraoperative brain relaxation and to facilitate surgical wound closure in the postoperative period.

Once the CPA was exposed, direct stimulation of the VS surface was performed routinely by using a monopolar stimulator, to identify any posterior dislocation of the VII cranial nerve. Subsequently, the tumor capsule was opened, and intralesional debulking was performed to reduce tumor size to identify the FN along its cisternal course. Debulking was performed with the use of ultrasonic aspirator (Sonopet^®^, Stryker) by constant direct stimulation of the FN to promptly identify the nerve close to the tumor. Once the tumor volume in the CPA was reduced, the FN was identified at its origin from the brainstem. At this point, a search for the FN proximal ST was performed by direct stimulation (*see section IONM*).

After identifying the FN at its origin, lesion debulking proceeded, and the tumor was dissected from the FN following the plane previously identified on the brainstem.

In cases where the plane of dissection was not identifiable and in cases of infiltration of the nerve by the tumor, a residual tumor was left attached to the nerve to preserve both its anatomical and functional integrity.

At the end of resection, direct stimulation of the nerve at its emergence to the brainstem was repeated and the new ST was recorded.

#### 2.2.1 IONM

All the procedures were performed using a neurophysiological monitoring system including motor evoked potentials (MEPs), sensory evoked potentials (SEPs), brainstem acoustic evoked potentials (BAEPs), free running-electromyography (EMG), and direct stimulation. In patients undergoing surgery in the last 2 years of the series, monitoring of corticobulbar evoked potentials for FN (f-MEPs) was also introduced; however, because of the heterogeneity of the available data, information on such monitoring was not included in this study.

Data were collected for direct stimulation performed with NIM 3.0 (*Nerve Monitoring System*, *Medtronic*) that allowed to record the activity of selected muscle groups by EMG monitoring. Electrodes for FN monitoring were inserted at the level of the orbicularis muscle of the eye and the orbicularis of the mouth of the side corresponding to the nerve of interest for two-channel monitoring.

Monitoring was performed by associating continuous monitoring of free-running EMG activity and by direct stimulation with a monopolar stimulator, to identify, in the different phases of the surgery, both the nerve stimulation thresholds (STs) and the course of the dislocated nerve during tumor removal.

The stimulation phases were performed as follows: (1) identification of the FN ST at its emergence from the brainstem (proximal ST); (2) direct supra-threshold stimulation during tumor debulking to identify and map the nerve along its course; (3) check of proximal ST before starting the intra-meatal removal phases; (4) check of proximal ST at the end of tumor removal.

Intraoperative neurophysiological monitoring (IONM) data collected and evaluated in the study were as follows: -*Facial nerve threshold T0 (FT-0)*: The minimum amplitude (milliamperes) required to obtain an EMG response of the nerve at its proximal emergence at the brainstem level before tumor removal; -*Facial nerve threshold T1 (FT-1)*: The minimum amplitude (milliampere) required to obtain an EMG response of the nerve at its proximal emergence at brainstem level after tumor removal; -*Delta threshold (DT)*: The difference in absolute value between FT-1 and FT-0.

### 2.3 Extent of resection

The extent of resection was defined by evaluating gadolinium-enhanced T1w MRI sequences performed at 3 months after surgery. GTR was defined as the complete absence of residual detectable on T1w MRI with gadolinium, NTR was defined as the presence of residual with a diameter ≤ 2 mm often not visible on MRI but left *in situ* during surgery to functionally preserve the nerve, and STR was defined as the presence of residual not attributable to NTR.

### 2.4 Outcome definition and classification

The primary outcome of the study was FN function after surgery and was assessed using the HB clinical scale (25). In this study, House–Brackmann (HB) scale values were used as the primary outcome and were grouped into two categories so that the analytic evaluation could be based on nominal variables. The categories used were “good” including patients with HB 1 and/or 2 and “poor” including patients with HB values of 3, 4, 5, and 6.

Clinical evaluation of FN function was performed by a team of three experienced neurosurgeons (RDM, ELB, and FZ) at different times during the postoperative period and was done by direct clinical interview and/or video call. Specifically, HB grade was assigned at postoperative day 4 (T1), at 3 months after surgery (T2), 12 months after surgery (T3), and during clinical evaluation at the last available follow-up (T4). Given the inherent subjectivity of the adopted scale, if there was discordance between the values assigned by the different examiners, then the worst score was considered.

### 2.5 Statistical analysis

Descriptive statistics were reported with mean and standard deviation for cardinal variables and with frequency and percentage for categorical variables. A Shapiro–Wilk Test was used to assess the normality of the distribution of quantitative variables and a Spearman’s Rho test to assess the concordance of scores for quantitative variables. The existence of a model with statistically significant predictive ability toward the dichotomized HB grade was assessed using the binary logistic regression model. Statistical significance was set at p ≤ 0.05. All the analysis was conducted on SPSS (version 26.0; IBM Corp., Armonk, NY).

## 3 Results

After a thorough evaluation of the inclusion and exclusion criteria, a total number of 72 patients were considered eligible for the study. Twenty-eight patients were not included because of the lack of facial function data at 1 year (15 patients), realization of facial-hypoglossal anastomosis (five patients), and a positive history of previous surgery and/or radiosurgery (eight patients).

Most of patients were women with a ratio of 2.4:1 [51 F (70.8%) and 21 M (29.2%)]. The mean age was 59.9 ± 8.76 years (p = 0.159). Regarding neuroimaging data, the mean largest tumor diameter was 27.4 ± 18.59 mm (p < 0.001), and most tumors did not have the cystic component [51 (70.8%) vs. 21 (29.2%)]. In accordance with Samii’s classification, the most represented type was T4A [25 patients (34.7%)]. The mean duration of surgery was 324.16 ± 89.3 min (p < 0.001), and the extent of resection was 32 GTR (44.4%), 30 NTR (41.6%), and 10 STR (13.9%). Mean follow-up was 22.4 ± 18.4 months. As for complications, four patients (5.5%) developed a CSF (Cerebrospinal fluid) fistula, which was subsequently surgically repaired, one patient (1.3%) developed hydrocephalus undergoing peritoneal ventricular shunt placement, and one patient (1.3%) developed transverse and sigmoid sinus thrombosis that was subsequently solved with heparin therapy.

**Figure 1:**
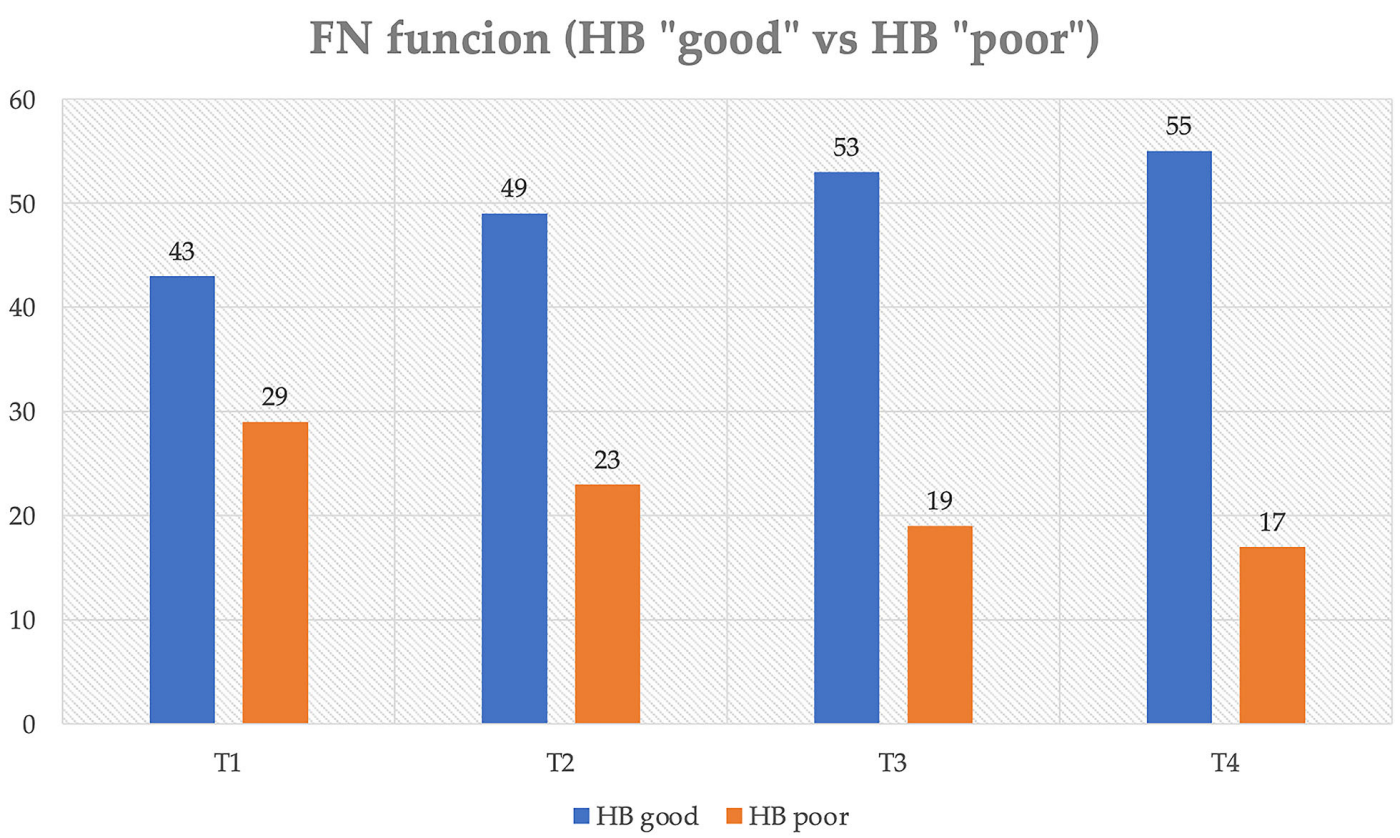
Distribution of postoperative FN function (House–Brackmann scale) over time. T1, IV postoperative day; T2, 3-month follow-up; T3, 1-year follow-up; T4, last follow-up.

Analyzing FN function according to HB, 43 patients belonged to the HB “good” group on postoperative day 4, 49 patients at 3 months, and 53 patients at 12 months. At the last follow-up, 76.4% of patients showed “good” HB versus 23.6% of patients with “poor” HB. Specifically, 43 patients (59.7%) had HB value of 1 at 12 months and at the last follow-up, whereas patients with HB value of 2 were 10 (13.9%) at 12 months and 12 (16.7%) at the last follow-up (**Figure 1**).

All population characteristics were reported in **Table 1**.

**Table 1:** Demographics: quantitative and qualitative variables.

Variable	Mean	SD	Shapiro–Wilk Test
Age	59.91	8.76	p = 0.159
Tumor major diameter	27.44	18.59	p < 0.001
Facial nerve stimulation T1 (mA)	0.20	0.35	p < 0.001
Delta threshold (mA)	0.18	0.35	p < 0.001
Surgical time (min)	324.16	89.33	p = 0.049

Variables		Frequencies	%
**Sex**	F	51	70.8
	M	21	29.2
**Cystic**	Yes	21	29.2
	No	51	70.8
**Samii grade**	T1	2	2.8
	T2	5	6.9
	T3a	16	22.2
	T3b	20	27.8
	T4a	25	34.7
	T4b	4	5.6
**Koos**	1	3	4.2
	2	23	31.9
	3	19	26.4
	4	27	37.5
**HB T1 (IV postop day)**	1	22	30.6
	2	21	29.2
	3	14	19.4
	4	10	13.9
	5	5	6.9
**HB T2 (3 months)**	1	33	45.8
	2	16	22.2
	3	9	12.5
	4	8	11.1
	5	6	8.3
**HB T3 (12 months)**	1	43	59.7
	2	10	13.9
	3	8	11.1
	4	6	8.3
	5	5	6.9
**HB T4 (last follow-up)**	1	43	59.7
	2	12	16.7
	3	9	12.5
	4	4	5.6
	5	4	5.6
**Extent of resection**	GTR	32	44.4
	NTR	30	41.6
	STR	10	13.9
**Complications**	CSF Leak	4	5.5
	Hydrocephalus	1	1.3
	Sinus thrombosis	1	1.3

A Shapiro–Wilk test was used to assess normality of distribution of quantitative variables.

### 3.1 Statistical analysis

First, a direct statistical association was investigated between variables, and, thereafter, a logistic regression model was built to analyze the relationships of these variables with the FN functionality during the follow-up. All the variables that had been found to be significantly correlated in the multivariate analysis models were merged to build a score (see section FNOS) after defining statistically significant cutoffs for continuous variables. Age, surgical time, and the extent of resection did not result to be significantly associated to postoperative HB grade.

Finally, the value resulting from the FNOS was assigned to all patients, and univariate and multivariate analyses were performed to validate the score, demonstrating a significant association with the outcome measure (FN function).

Initially, a statistically significant linear correlation was observed between HB scale values and different assessment timings (p < 0.01). As shown in **Supplementary Table 2**, there was a strong statistically significant association (V = 0.774, p < 0.01) between the HB grade recorded at IV postoperative day (T1) and 3- month follow-up (T2). Only 2.3% of patients in the group of “good” HB (HB < 3) at T1 experienced a worsening at T2, whereas the 24.1% of “poor” HB subjects at T1 had an improvement at T2. Similarly, these associations were confirmed between the degree of HB at T1 and T4 (V = 0.677, p < 0.01). None of the “good” HB subjects at T1 had a worsening at T4; 41.4% of the “poor” HB subjects at T1 had an improvement at T4.

An ordinal logistic regression model using the degree of HB (HB < 3 vs. ≥ 3, “good” vs. “poor”) showed a statistically significant association with the maximum tumor diameter, Samii grade, the F-T1, and the DT.

Specifically, the absence of a cystic component was found to be a protective factor. Indeed, the likelihood of observing an elevated HB value was, respectively, −2.6- and −1.3- fold lower in solid tumors both in the medium and long terms [T2: Nagelkerke R-square, 0. 603 with p < 0.05; Exp(B), 2.60; p < 0.001; T3: R-square Nagelkerke, 0.645 with p < 0.05; Exp(B), 1.28; p < 0.001; T4: R-square Nagelkerke, 0.598 with p < 0.05; Exp(B), 1.29; p < 0.001]. Similarly, DT appeared to be a worsening factor with an approximately seven-fold greater likelihood at T2 and T3 and approximately six-fold greater likelihood at T4 of observing an elevated HB value [T2: R-square Nagelkerke, 0. 603 with p < 0.05; Exp(B), 7.03; p < 0.001; T3: R-square Nagelkerke, 0.645 with p < 0.05; Exp(B), 7.10; p < 0.001; T4: R-square Nagelkerke, 0.598 with p < 0.05; Exp(B), 5.79; p < 0.001]. Last, FT-1 showed a strong relationship in the logistic regression analysis, resulting in a seven-fold increase at T2 and a five-fold increase at T3 in the risk of obtaining a “poor” HB for each increase in FT-1 (**Table 2**).

**Table 2:** Multivariate analysis showing the role of different variables influencing FN outcome at different time (T2–T4).

T2			
Nagelkerke R^2^	Model’s Fit	Goodness of Model	
		Pearson	Variance
0.603	p < 0.05	p > 0.05	p > 0.05
Variable		Exp(B)	p-Value
Samii Grade		17.18	**0.000**
Cystic		−2.60	**0.001**
DT		7.035	**0.000**
FT-1		7.0	**0.001**

T3			
Nagelkerke R^2^	Model’s Fit	Goodness of Model	
		Pearson	Variance
0.645	p < 0.05	p > 0.05	p > 0.05
Variable		Exp(B)	p-Value
Samii grade			–
Cystic		−1.28	**0.001**
DT		7.10	**0.000**
FT-1		5.0	**0.007**

T4			
Nagelkerke R^2^	Model’s Fit	Goodness of Model	
		Pearson	Variance
0.603	p < 0.05	p > 0.05	p > 0.05
Variable		Exp(B)	p-Value
Samii grade			–
Cystic		−1.287	**0.001**
DT		5.79	**0.000**
FT-1		1.7	**0.01**

The bold values denote statistical significance a P < 0.05 level.

The univariate (**Supplementary Table 3**) and the multivariate analyses with binary logistic regression using as dependent variable the HB “good” and HB “poor” groups confirmed the statistical significance of the independent variables found to be associated in the previous model.

As next step, cutoffs were identified by descriptive analysis of statistically significant associations. Although association analysis was performed at various times, because of the high association value identified at 12 months (p < 0.001), cutoffs were identified at T3 for both the DT and FT-1 variables (**Supplementary Table 4**).

Indeed, 100% of patients with DT ≤ 0.07 and 66.7% of patients with DT between 0.07 and 0.19 were HB “good” at 3 months, whereas 100% of patients with DT > 0.19 had HB “poor” at the same time (Cramer’s V, 0.896; p < 0.001). Similarly, statistically significant associations between FT-1 value and HB grade group were reported in (**Supplementary Table 3**). Specifically, 100% of patients with FT-1 ≤ 0.08 and 70% of patients with FT between 0.08 and 0.20 were HB “good” at 3 months, whereas 100% of patients with FT > 0.20 had HB “poor” (Cramer’s V, 0.892; p < 0.001).

Once cutoffs of continuous variables and significantly associated categorical variables were identified, the relationship between FNOS and HB groups (“good” vs. “poor”, < 3 vs. ≥ 3) was assessed.

### 3.2 Facial Nerve Outcome Score

**Figure 2:**
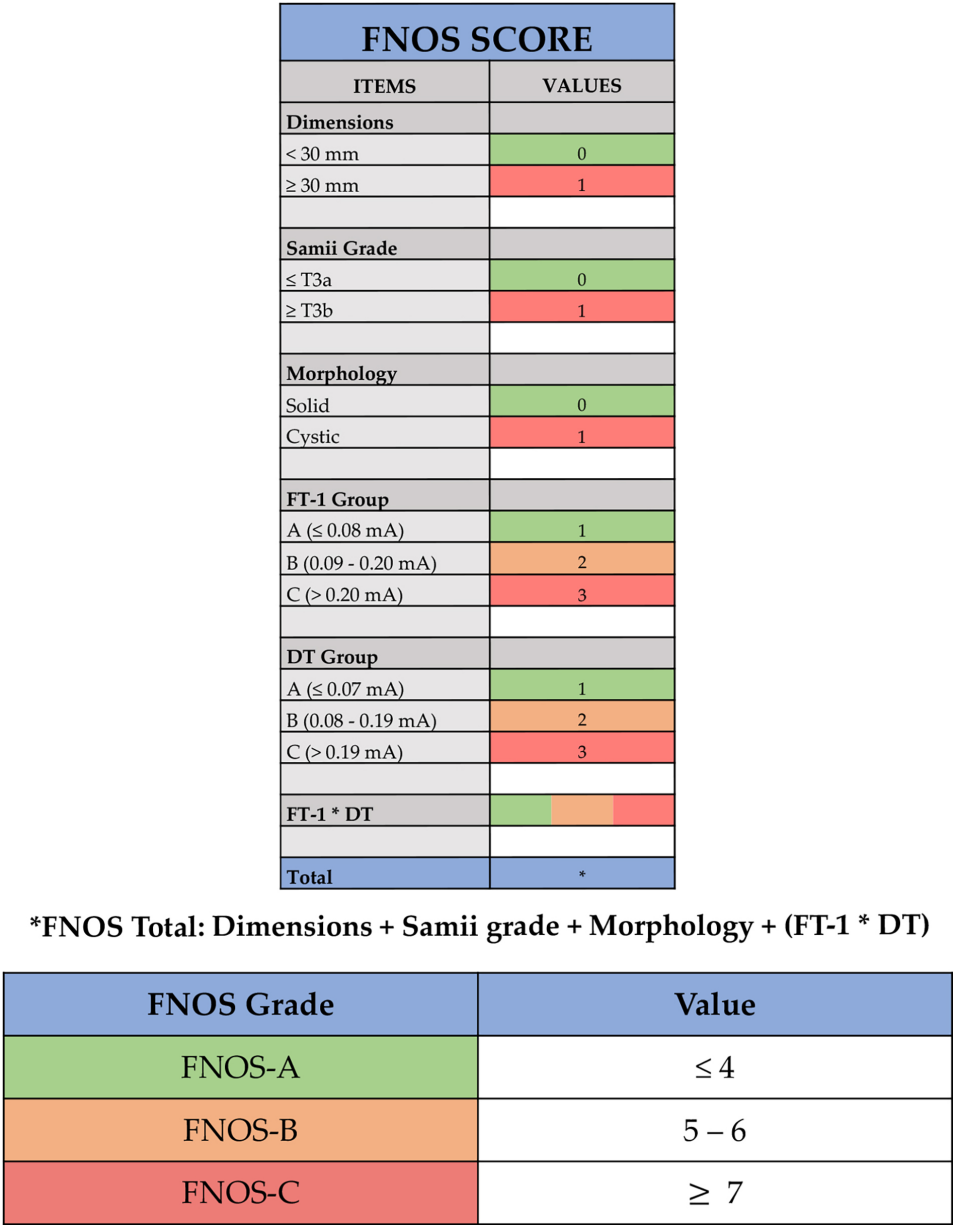
Facial Nerve Outcome Score (FNOS). Each item of the score is showed: dimensions, Samii grade, morphology, facial nerve threshold T1 (FT-1) (the minimum amplitude (mA) required to obtain an EMG response of the nerve at its proximal emergence at brainstem level after tumor removal), and delta threshold (DT) (the difference in absolute value between FT-1 and FT-0). The final score is the result of dimensions + Samii grade + morphology + (FT-1 * DT).

Building the score was based on the idea of being able to interpolate the statistically correlated independent variables with the postoperative FN function. Tumor size, Samii classification, solid or cystic radiological aspect, FT-1, DT, and FT-1 * DT all showed a statistical significance in the logistic regression and were retrieved as independent variable of the score (**Figure 2**).

In terms of cutoff, an arbitrary threshold of 30 mm was defined considering the average value of study population (27 ± 18 mm) and the results in terms of FN function obtained for tumors with a maximum diameter greater than 30 mm (21, 22, 26–30).

The Samii grade and dimensions appeared to be related statistically to the FN function at FU. T3a Samii grade, namely, a tumor extending in the CPA cistern and in contact with the trunk, causes a more important dislocation of the FN, making its surgical dissection more difficult and at higher risk of postoperative deficit (22, 27, 31, 32). For this reason, one point was given in case of tumor presenting as Samii T3b or T4.

One point was given for the presence of intratumor cystic component, because of its absence resulted to be a protective factor, reducing by about two times the probability of observing a deficit of FN HB “poor”.

Three ranges were identified for the maximum nerve ST at its brainstem emergence recorded at the end of surgery (FT-1), assigning one point for values of FT-1 < 0.08 mA, two points for values of FT-1 between 0.09 mA and 0.20 mA, and three points for values of FT-1 > 0.20 mA.

Similarly, the difference between FT-1 (at the end) and FT-0 (at the beginning) (DT) was divided in values < 0.07 mA (one point), values between 0.08 and 0.19 mA (two points), and values > 0.19 mA (three points).

Given the close mathematical correlation of the parameters DT and FT-1, the value considered by the score for the calculation of the final score did not take into account the individual values but the product of both, reported in the score as FT-1 * DT.

The final score was the result of the sum of the individual scores assigned to the following four items: tumor size (< 30 mm and ≥ 30 mm), Samii classification (≤ T3a and > T3b), morphology (solid and cystic), and FT-1 * DT.

According to the result, ranging from 1 to 12, three groups of patients can be identified:

-FNOS-A for scores ≤ to 4;
-FNOS-B for scores between 5 and 6;
-FNOS-C for scores ≥ to 7.

**Figure 3:**
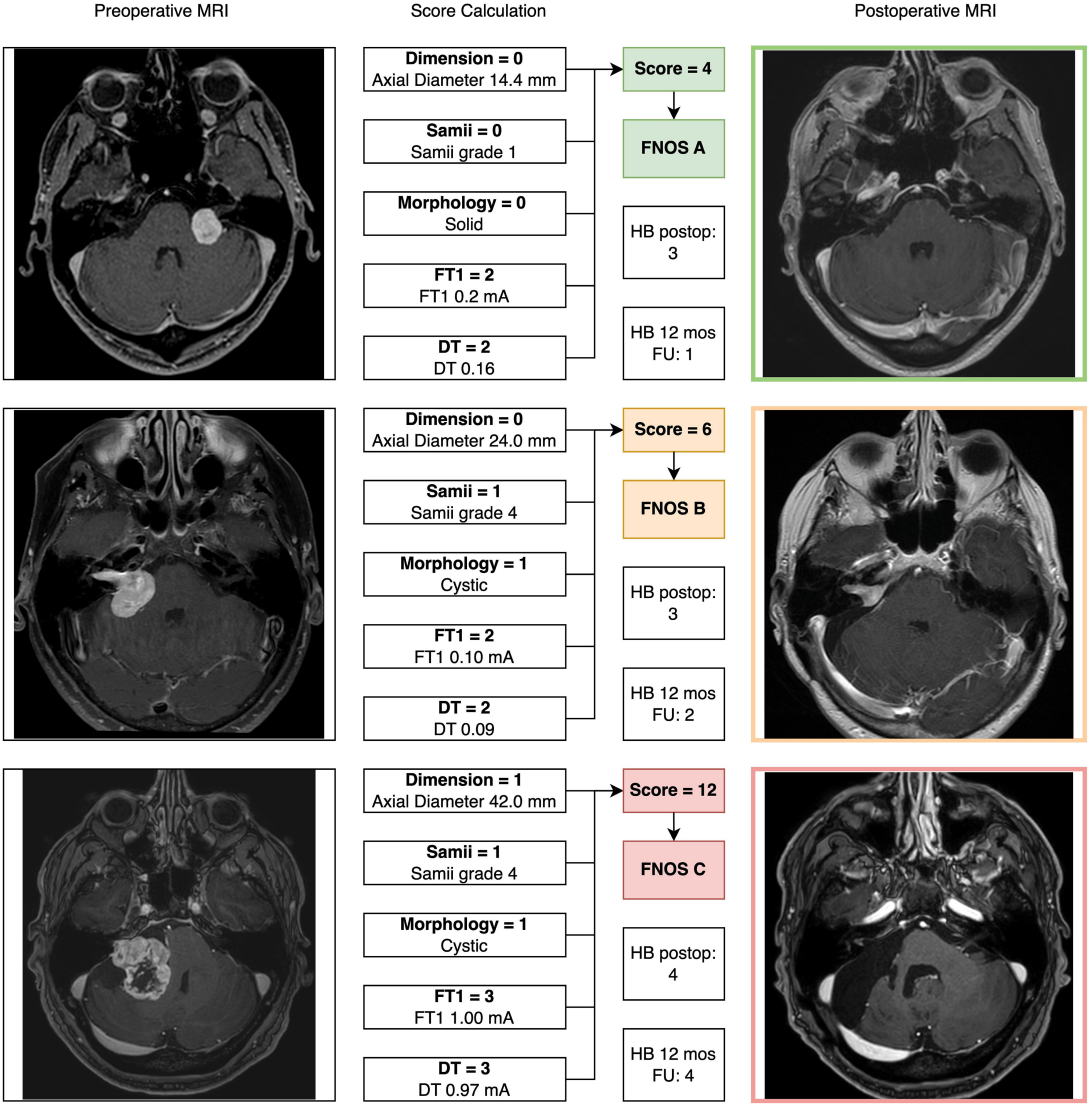
Three exemplary cases of patients with vestibular schwannoma were reported illustrating the “Facial Nerve Outcome Score” calculation. In all cases, a 12-month postoperative brain MRI (axial cut of T1-weigthed contrast enhanced MRI) showed the extent of resection.

Three cases illustrating score calculation are reported in **Figure 3**.

**Figure 4:**
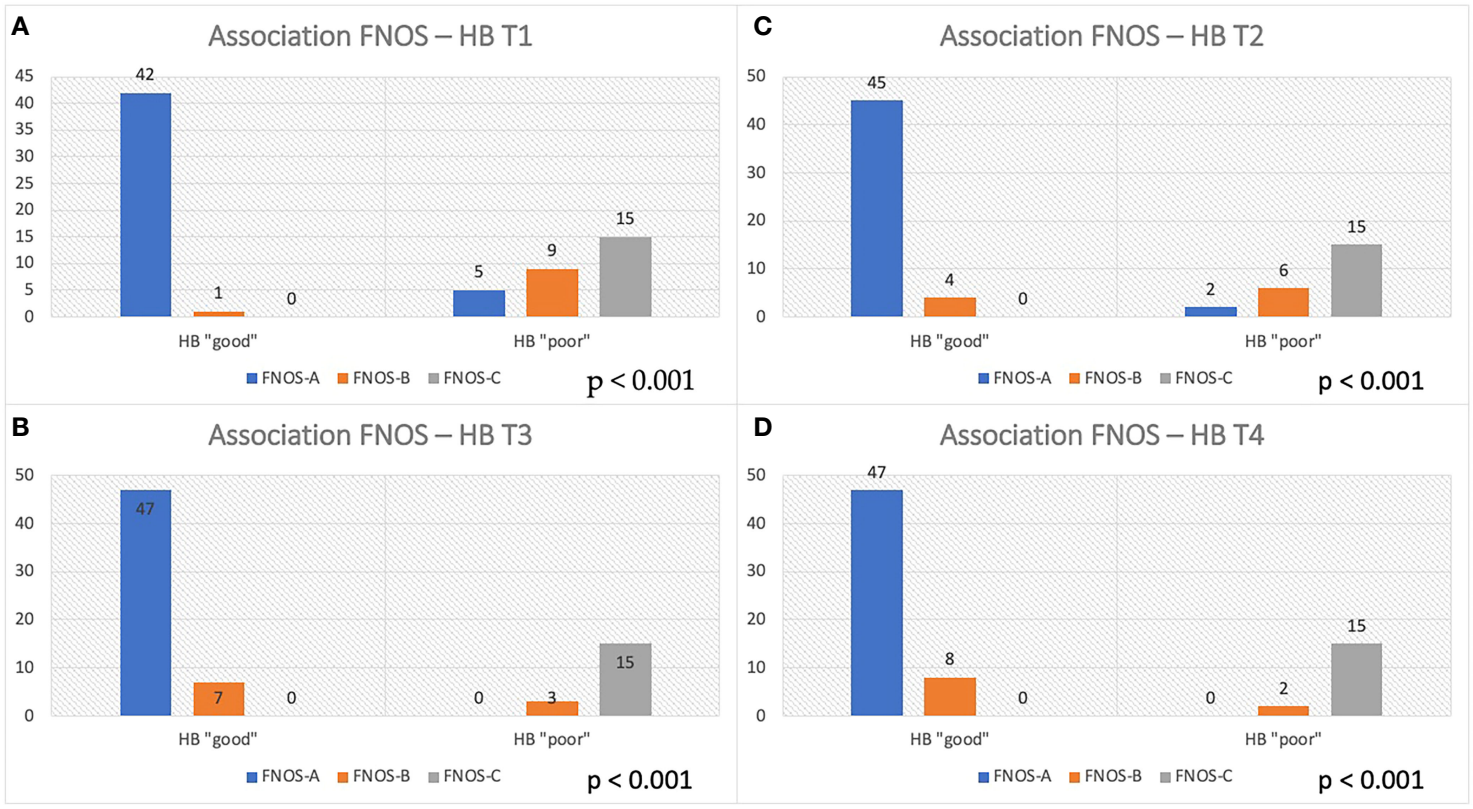
Association between FNOS and FN function (HB grade) at different time (T1-T4, **A–D**). Patients are divided in "good" and "poor" HB outcome < 3 and ≥ 3, respectively, considering the result of the score FNOS (FNOS-A, -B and -C).

At this point, an association was investigated between the FNOS grade the HB group at different moments of the FU. A strong and increasing positive association between FNOS grade and HB grade was observed at both T1, T2, T3, and T4 (**Tables 3A-D**; **Figure 4**). However, this association was more evident at 12 months and at the last follow-up (T1 Cramer’s V, 0.831; T2 Cramer’s V, 0.851; T3 Cramer’s V, 0.919; T4 Cramer’s V, 0.936; p < 0.001). Specifically, 100% of patients with FNOS-A belonged to HB “good” group at 12 months after surgery, whereas decreasing at 70% for those with FNOS-B and 100% of patients with FNOS-C presented an HB ≥ 3 at 12 months (**Table 3**).

**Table 3:** Association between FNOS class and FN function (HB grade) at different time (T1–T4).

a			
Variable		HB T1	
	Modality	HB “good”	HB “poor”
**FNOS**	A ≤ 4	42	5
	%	89.4	10.6
	B = 5–6	1	9
	%	10	90.0
	C ≥ 7	0	15
	%	0	100
Chi-Squared		Cramer’s V	p-Value
49.684		0.831	**0.000**

b			
Variable		HB T2	
	Modality	HB “good”	HB “poor”
**FNOS**	A ≤ 4	45	2
	%	95.7	4.3
	B = 5–6	4	6
	%	40	60
	C ≥ 7	0	15
	%	0	100
Chi-Squared		Cramer’s V	p-Value
60.800		0.851	**0.000**

c			
Variable		HB T3	
	Modality	HB “good”	HB “poor”
**FNOS**	A ≤ 4	47	0
	%	100	0
	B = 5–6	8	2
	%	80	20
	C ≥ 7	0	15
	%	0	100
Chi-Squared		Cramer’s V	p-Value
60.364		0.919	**0.000**

d			
Variable		HB T4	
	Modality	HB “good”	HB “poor”
**FNOS**	A ≤ 4	47	0
	%	100	0
	B = 5–6	9	4
	%	81.8	18.2
	C ≥ 7	0	15
	%	0	100
**Chi-Squared**		Cramer’s V	p-Value
63.129		0.936	**0.000**

Patients were divided in “good” and “poor” HB outcome (< 3 and ≥ 3, respectively).

The bold values denote statistical significance a P < 0.05 level.

Finally, a binary logistic regression confirmed the existence of this statistically significant association of the score with the HB group with a model with good reliability at 3 and 12 months, whereas losing statistical significance at the last follow- up for the violation of the assumptions of logistic regression, given the high strength of association (**Supplementary Table 5**). On an in-depth view, the unitary increase of FNOS value showed an increase by about three times the probability of observing a “poor” HB value at 3 months [R-square Nagelkerke, 0.798; Exp(B), 2.999; p < 0.001] and by about five times at 12 months [R-square Nagelkerke, 0.891; Exp(B), 5.486; p < 0.001].

Similarly, the binary logistic regression analysis performed using the categorical FNOS variable to assess the probability of observing a “good” or “poor” HB value at T2, T3, and T4 lost statistical significance because the high precision of the model violated the regression axioms (see percentages of model accuracy in **Supplementary Table 5**).

## 4 Discussion

Important advances in the treatment of VS have been registered in recent years due to technological development and the growth of surgical technique. This improvement has increasingly highlighted the importance of functional preservation of the FN and overall neurological status (30, 33–35).

Several studies reported percentages ranging from 60% to 90% of patients with HB 1 or 2 at 12 months after surgery, emphasizing the importance of several clinical, morphological, and functional factors significantly correlated with this outcome (21, 29, 30, 36).

In the present study, the percentage of patients with FN function classifiable as “good”, HB < 3, resulted 73.7% at 12 months after surgery, reaching 76.4% at the last follow-up (mean, 24 months). Tumor size, tumor morphology, the grade according to the Samii classification, and FT-1 and DT, among IONM parameters, were found to be significantly related to the long-term FN function. Meanwhile, unlike other studies, the extent of resection was not statistically significant. These factors represent the structural pillars of the FNOS, a score that combines the importance of neurophysiological parameters of FN function with tumor characteristics and was found to be strongly associated with long-term FN function.

### 4.1 Tumor size

Since the first studies concerning VS surgery, tumor size, predominantly assessed as the largest diameter of the cisternal component of the tumor, has been found to be a prognostic factor related to postoperative FN function (22, 26–28), although there is no homogeneity in the definition of a true size cutoff (20).

A grater diameter means often a pre-existing nerve suffering due to compressing and stretching forces exerted by large tumors and the need for more aggressive and repeated surgical maneuvers. Those were the explanation of the higher 1-year incidence of patients with HB greater than 3 among stage IV tumors (45% HB value of 3–6) reported by Rinaldi et al. (28). In the present study, tumor size was not significantly associated with HB grade in the long term (p = 0.100) but maintained significance of the association until T2 (p = 0.05). The latter was a result that it is not too different from another one registered in 256 patients (30) although the maximum diameter of involved tumors was 15 mm.

### 4.2 Grade according to Samii

Samii et al. in 1997, reporting their experience on the treatment of 1,000 VSs, proposed a grading scale and demonstrated how it correlated with facial nerve function (22). The important role of this grading system in predicting FN outcome was more recently confirmed in 212 patients as well (32); the percentage of patients with HB 1 to 3 was 90% for Samii grade 2–3 tumors (T2–T3) versus 70% of tumors classified as T4 (p < 0.001). Moreover, in addition to the relationship between Samii grade and the anatomical and functional preservation of the facial nerve, the FN outcome could rely on the type of nerve displacement. Indeed, the anterior dislocation of the nerve led to significantly better results (HB I and II: 76%) compared to antero-lateral or antero-medial dislocation (HB I and II: 60%).

This aspect, replicated in the literature (21), could be related to the type of relationship that the tumor develops with the trunk, considering that the dislocation of the trunk can alter the position of the nerve emergence in regard to its entrance in the internal auditory meatus, causing its rotation along its major axis in the cisternal tract, where, together with fibers splitting induced by the tumor, it could lead to the presence of greater antero-superior and antero-inferior displacement in the Samii T4 grades.

**Figure 5:**
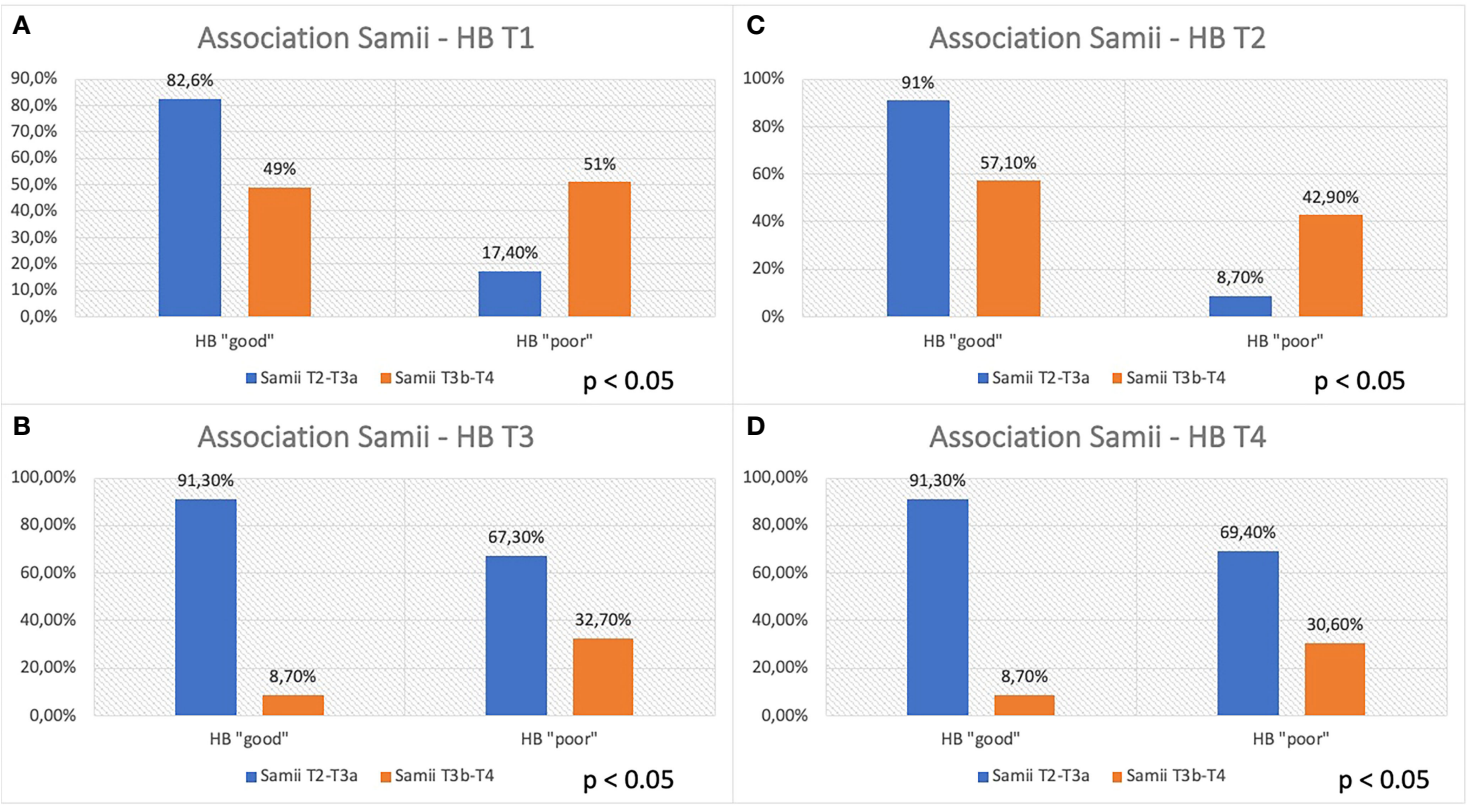
Association between Samii grade and FN function at different time (T1–T4, **A–D**). Patients are divided, considering the Samii grade (T2–T3a vs. T3b–T4), in “good” and “poor” HB outcome, < 3 and ≥ 3, respectively.

In the present study, although nerve dislocation pattern was not considered, the percentage of patients with “good” HB in Samii T4 tumors was significantly lower than in patients with Samii T2 and T3 tumors both in the short and long terms (HB “good” at 12 months: 93%, Samii T2–T3; 67%, Samii T4; **Figure 5**). Moreover, this difference is even more marked in the short-term (HB “good”: 17% postoperatively and 8% at 3 months in Samii T4 tumors), confirming that may be an effect strictly connected to surgical maneuvers but not exclusively relied on them, giving the presence of other factors that can affect the long-term outcome (i.e., functional status of the nerve and type of nerve damage).

### 4.3 Morphology

Several studies in the literature highlighted the importance of the presence of a cystic component within the VS and the influence that this component may have on the FN function (21, 37–40).

Moon et al., analyzing 106 VSs (24 cystic and 82 solids), reported a higher incidence of nerve sections and worse function according to HB grade in patients with cystic VSs. The authors also hypothesized the role played by the expression of matrix metallo proteases (MMPs). In particular, the high expression of MMP-2 within the cyst fluid and the inner layer of the cyst wall would seem to be responsible for the tumor size enlargement and the marked adhesion of cystic VS to the nerve, also emphasizing the role that the proteolytic activity of MMP-2 may play on the integrity of the blood-liquid barrier at the nerve surface (39).

Although many works in the literature agree on the increased risk of nerve damage and recommend the observance of some technical expedients such as blunt dissection, considering the absence of a true arachnoid plane with the cyst wall, some authors did not find a statistically significant difference on FN function between solid and cystic VSs (32, 41).

In the present study, the presence of a cystic component was significantly correlated with FN function in logistic regression analysis. Specifically, the absence of a cystic component results in an approximately two-fold reduced probability of having a “poor” HB at postoperative and at 3 months. When evaluated at 12 months and at the last follow-up, this correlation— always significant —is reduced to a probability of having a “poor” HB grade about one time lower. This aspect underlines once again how the impact of factors related to the morphology of the tumor seems to have a greater predictive power in the short term than in the long term, probably because of surgical maneuvers.

### 4.4 FT-1 * DT

In the increasingly demonstrated surgical perspective of favoring FN preservation over the extent of resection of VSs, shifting the goal from maximal resection to maximal “safe” resection, scientific interest in IONM and various techniques for intraoperative monitoring of FN function has exponentially grown (15, 17, 18, 24, 30, 32, 34, 36, 41–46).

Since the important experience of Goldbrunner et al. reporting an increase from 1.6% to 75% in the probability of having a significant facial deficit at 6 months for patients with proximal-distal EMG ratios < 0.8 and < 0.128, respectively (16), different parameters of IONM have been investigated obtaining good results. Indeed, Prell et al. found a “poor” FN outcome for A-train times greater than 10 s (44), Lin et al. found a positive predictive value in stimulating the nerve emergence at level of the brainstem and evaluating the EMG response in microV (18), and others found a predictive value even more significant combining two or more variables (43).

More recently, the use of FN cortico-bulbar evoked potentials (f-MEPs) has been introduced to increase the predictive power of the IONM (9, 47). A statistically significant correlation was found between f-MEPs with HB grade at 6 weeks, 6 months, and 12 months after surgery (78% and 73% of HB I or II, respectively) (36, 46).

A strong association between intraoperative FN stimulation parameters and short- and long-term postoperative HB grade in both univariate and multivariate analyses was confirmed in the current results (**Supplementary Table 4**). A total of 100% of patients with FT-1 ≤ 0.8 mA and DT ≤ 0.7 mA reported “good” HB at 12- month follow-up and conversely at the same time 100% of patients with FT-1 > 0.20 mA and DT > 0.19 mA reported “poor” HB, whereas, for intermediate values of FT-1 (0.09–0.20 mA) and DT (0.08–0.19 mA), the percentages of “good” HB at 12 months were 69.2% and 66.7%, respectively.

### 4.5 Score strength and gray zone

The FNOS was found to be a reliable tool with a strong statistical association with postoperative FN function in both the short- and long-term. Ordinal logistic regression analysis showed that, for each one-point increase in the score, there was a significant approximately five-fold increase in the risk of obtaining a “poor” HB outcome at 1-year after surgery, underscoring the importance of the parameters that constitute the score.

A total of 100% of FNOS-A patients (scores 1–4) have “good” HB at 12 months and at last follow-up. Conversely, 100% of FNOS-C patients (scores 7–12) have “poor” HB at 12 months and at last follow-up. Although the strength of association of the score remains high even in the short term, it was lower than in the long term (Cramer’s V T3/T4 of 0.919/0.936 vs. Cramer’s V T1/T2 of 0.831/0.851), being, indeed, significantly lower the percentages of patients with HB “good” in the postoperative and at 3 months for FNOS-A patients (T1, 89.4%; T2, 95.7%). The reason behind could be two-fold: The stimulation parameters have demonstrated a greater influence on the FN function on the long-term, whereas the morphological parameters have shown a greater strength on the short term. Probably, this aspect could be related to the fact that, in the first 3 months after surgery, the surgical maneuvers related to the size, morphology, and relationships of the nerve with adjacent structures play a more important role on the nerve damage. Furthermore, it could be hypothesized that the nerve damage in these patients is predominantly neuropraxic, considering the percentage of FNOS-A patients with “good” HB grade on the long-term follow-up (**Figure 4**).

The latter aspect is more stressed in patients who fall into a “gray zone” of the score, namely, FNOS-B patients.

As shown in **Figure 4**, these patients show higher percentages of “HB poor” up to 3 months after surgery and then show satisfactory percentages of “good” HB in the long-term, underlining the ability of the score to intercept the extent of FN damage and consequently the potential for recovery (HB “poor” FNOS B: T1, 90%; T2, 60%; T3, 20%; and T4, 18.2%). These data represent an important aspect regarding the fact of being able to expect a long-term improvement in patients with postoperative FNOS-B who present a grade HB “poor” in the short-term follow- up. In these patients, it is likely that the nerve damage is predominantly in the sphere of axonotmesis or that there may be factors —already described in the literature— not strictly related to surgery, that may affect the short-term nerve function (e.g., neuroinflammation, vasospasm, and herpes simplex virus reactivation) (22, 48, 49). Nevertheless, although strong association was found, the small size of this sample did not allow to adequately assess the predictive value of the gray zone.

## 5 Limitations

One of the main limitations of the study is linked to its retrospective nature, although the prospectively collected data allow for adequate statistical analysis. Unfortunately, the small size of the cohort decreases the statistical power of the result and of the predictive value of the score. Furthermore, the IONM parameters used are only direct nerve stimulation parameters, and this represents a limitation, given the increasingly use of f-MEPs and their scientific validation, associated with the possibility of overcoming limitations arising from EMG alone (i.e., the repeatability of f-MEPs during tumor removal even without direct nerve exposure).

Another important aspect is the violation of regression assumptions for the analysis of the categorical variable FNOS. This violation, due to the high association obtained from the score with the outcome on the HB scale, represents a statistical limitation to the possibility of building a probabilistic predictive model that allows to estimate the exact likelihood of having a “good” or a “poor HB” on the basis of belonging to each FNOS group. However, binary logistic regression analysis with the FNOS scores on an ordinal scale allows us to significantly estimate the probability of having a more or less good outcome for each one-point change in the score. This seems to be mainly related, in part, to the relatively small number of patients but mainly to the discrepancy between the higher number of patients with a good outcome compared with those with a bad outcome. Thus, because of the low variance, it was impossible to obtain a statistically significant predictive model of the FNOS score, although the association of the score with HB class in the long term was well established. On the other hand, a higher percentage of favorable outcomes represents the goal of surgery, and the statistical need to analyze more negative outcomes would create a problem from an ethical point of view.

Therefore, if, on the one hand, statistically speaking, this represents the major limitation of the study; on the other hand, from a clinical and research point of view, it could represent a solid and concrete starting point for further research. Indeed, by increasing the number of patients and considering the multicentric extension of the analysis, it is expected that the predictivity of the individual classes of the score could reach statistical significance due to the greater number of variables included and the proportional increase of unfavorable outcomes.

## 6 Conclusions

The FNOS represents a reliable score and resulted to be strongly associated with long- term FN function. A one-point score increase showed to possibility to predict a five-fold increase of the risk of “poor” HB at 12 months after surgery. Direct nerve stimulation parameters play a crucial role in predicting long-term facial function. The percentage of patients belonging to the gray zone (FNOS-B) with a FN function classifiable as HB “poor” in the short term significantly decreases in the long term, suggesting the potential ability to catch patients with higher chance for recovery that should be better assessed with larger and multicentric series.

## Data availability statement

The original contributions presented in the study are included in the article/**Supplementary Material**. Further inquiries can be directed to the corresponding author.

## Ethics statement

An informed consent was signed for clinical and surgical procedures. Ethical review and approval was not required due to the retrospective nature of the study.

## Author contributions

GDP: conceptualization, methodology, writing (original draft), and supervision; RDM: investigation, visualization and writing (review and editing); BMB: investigation and writing (review and editing); ELB: data collection; FC: writing (review and editing) and supervision; PZ: data collection; LC: formal analysis; FP: data collection; AM: writing (review and editing); DG: supervision; MML: supervision; FZ: supervision and conceptualization. All authors contributed to the article and approved the submitted version.
