# Supplementary material for: Facial nerve outcome score: a new score to predict long-term facial nerve function after vestibular schwannoma surgery
Source: Front Oncol. 2023 Jun 12;13:1153662. doi: 10.3389/fonc.2023.1153662 (PMC10291180; doi:10.3389/fonc.2023.1153662)
Supplement: Supplementary file 1 [file Table_1.docx]

Supplementary Material

Facial Nerve Outcome Score: A new score to predict long-term facial nerve function after vestibular schwannoma surgery

Giuseppe DI PERNA, MD^1,3,4^, Raffaele DE MARCO, MD^1,3*^, Bianca Maria BALDASSARRE, MD^1,3^, Enrico LO BUE, MD^1,3^, Fabio COFANO, MD^1,5^, Pietro ZEPPA, MD^1^, Luca CERONI^6^, Federica PENNER, MD^1,3^, Antonio MELCARNE, MD^1,2^ Diego GARBOSSA, MD, PhD^1,2^, Michele Maria Rosario LANOTTE, MD, PhD^1,7^, Francesco ZENGA, MD^2,3^

^1^Department of Neuroscience “Rita Levi Montalcini”, University of Turin, Turin, 10126, Italy

^2^Neurosurgery Unit, “Città della Salute e della Scienza” University Hospital, Turin, 10126, Italy

^3^Skull base and Pituitary Surgery Unit, “Città della Salute e della Scienza” University Hospital, Turin, 10126, Italy

^4^Casa di cura Clinica “Città di Bra”, Bra, Cuneo, 12042, Italy

^5^Spine Surgery Unit, Humanitas Gradenigo Hospital, Turin, Italy

^6^Department of Psychology, University of Turin, Turin, 10126, Italy

^7^Functional, Oncological and Stereotactic Neurosurgery Unit, “Città della Salute e delle Scienza” University Hospital, Turin, 10126, Italy

*** Correspondence:**Raffaele De Marco, MD

Neurosurgery Unit,

Department of Neuroscience “Rita Levi Montalcini”, University of Turin,

“Città della Salute e della Scienza” University Hospital,

Via Cherasco, 15, 10126, Turin, Italy

Tel: +393890120974

e-mail: [r_dema@outlook.it](mailto:r_dema@outlook.it); raffaele.demarco@unito.it

# Supplementary Tables

| **Inclusion criteria** | **Exclusion criteria** |
| --- | --- |
| - Age ≥ 18 years - Sporadic VS - Minimum follow up: 12 months - Presence of specialized mixed team - Availability of complete clinical, radiological, intra and post-operative data | - Diagnosis of NF 2 - Previous radiosurgery - Re-do surgery - Pre-operative FN disfunction - Intra-operative FN transection - Hypoglossal-facial nerves anastomosis |

**Supplementary Table 1.** Inclusion and exclusion criteria for retrospective analysis.

| **a** | | | | | |
| --- | --- | --- | --- | --- | --- |
| Variable |  | | **HB T2** | | |
| **HB T1** | Modality | | “Good” | | “Poor” |
|  | “Good” | | 42 | | 2 |
|  | % | | 97.7 | | 2.3 |
|  | “Poor” | | 7 | | 33 |
|  | % | | 24.1 | | 75.9 |
| **Chi Squared** | | **Cramer’s V** | | **pValue** | |
| 43.081 | | 0.774 | | **0.001** | |
| **b** | | | | | |
| Variable |  | | **HB T3** | | |
| **HB T1** | Modality | | HB “good” | | HB “poor” |
|  | HB “good” | | 42 | | 1 |
|  | % | | 97.7 | | 2.3 |
|  | HB “poor” | | 11 | | 18 |
|  | % | | 37.9 | | 26.4 |
| **Chi Squared** | | **Cramer’s V** | | **pValue** | |
| 31.821 | | 0.665 | | **0.001** | |
| **c** | | | | | |
| Variable |  | | **HB T4** | | |
| **HB T1** | Modality | | HB “good” | | HB “poor” |
|  | HB “good” | | 43 | | 0 |
|  | % | | 100 | | 0 |
|  | HB “poor” | | 12 | | 17 |
|  | % | | 41.4 | | 58.6 |
| **Chi Squared** | | **Cramer’s V** | | **pValue** | |
| 32.998 | | 0.677 | | **0.001** | |
| **d** | | | | | |
| Variable |  | | **HB T3** | | |
| **HB T2** | Modality | | HB “good” | | HB “poor” |
|  | HB “good” | | 49 | | 0 |
|  | % | | 100 | | 0 |
|  | HB “poor” | | 4 | | 19 |
|  | % | | 17.4 | | 82.6 |
| **Chi Squared** | | **Cramer’s V** | | **pValue** | |
| 54.989 | | 0.874 | | **0.001** | |
| **e** | | | | | |
| Variable |  | | **HB T4** | | |
| **HB T2** | Modality | | HB “good” | | HB “poor” |
|  | HB “good” | | 49 | | 0 |
|  | % | | 100 | | 0 |
|  | HB “poor” | | 6 | | 17 |
|  | % | | 26.1 | | 73.9 |
| **Chi Squared** | | **Cramer’s V** | | **pValue** | |
| 43.081 | | 0.811 | | **0.001** | |
| **f** | | | | | |
| Variable |  | | **HB T4** | | |
| **HB T3** | Modality | | HB “good” | | HB “poor” |
|  | HB “good” | | 53 | | 0 |
|  | % | | 100 | | 0 |
|  | HB “poor” | | 2 | | 17 |
|  | % | | 10.5 | | 89.5 |
| **Chi Squared** | | **Cramer’s V** | | **pValue** | |
| 62.075 | | 0.929 | | **0.001** | |

**Supplementary Table 2.** Associations between different binomial variables "HB group" at different assessment times (T1, T2, T3, T4).

| **Variable** | **Nagelkerke R^2^** | **Exp B** | **pValue** |
| --- | --- | --- | --- |
| **Samii** | 0.21 | 2.49 | **< 0.01** |
| **Cystic** | 0.35 | 13.43 | **< 0.01** |
| **Delta Threshold** | 0.76 | 2.32 | **< 0.01** |
| **Age** | 0.00 | 1.003 | 0.90 |
| **Koos grade** | 0.23 | 3.08 | **< 0.01** |
| **Second surgery** | 0.31 | 4.57 | 0.22 |
| **Extent of resection** | 0.008 | 1.37 | 0.53 |
| **Tumor size (maximum diameter)** | 0 | 1.002 | 0.90 |

**Supplementary Table 3.** Univariate analysis.

| Variable |  | **HB T3** | |
| --- | --- | --- | --- |
|  | Modality | HB “good” | HB “poor” |
| **Cut-offs FT-1** | **≤ 0.08** | 45 | 0 |
|  | % | 100 | 0 |
|  | **0.09 – 0.20** | 9 | 4 |
|  | % | 69.2 | 30.8 |
|  | **> 0.20** | 0 | 14 |
|  | % | 0 | 100 |

| **Chi Squared** | **Cramer’s V** | **pValue** |
| --- | --- | --- |
| 57.231 | 0.892 | **0.000** |

| Variable |  | **HB T3** | |
| --- | --- | --- | --- |
|  | Modality | **HB “good”** | **HB “poor”** |
| **Cut-offs DT** | **≤ 0.07** | 46 | 0 |
|  | % | 100 | 0 |
|  | **0.08 – 0.19** | 8 | 4 |
|  | % | 66.7 | 33.3 |
|  | **> 0.19** | 0 | 14 |
|  | % | 0 | 100 |

| **Chi Squared** | **Cramer’s V** | **pValue** |
| --- | --- | --- |
| 57.778 | 0.896 | **0.000** |

**Supplementary Table 4.** Cut-off definition for FT-1 and DT.

| **HB T2** | **Nagelkerke R^2^** | **Exp(B)** | **pValue** |
| --- | --- | --- | --- |
| FNOS | 0.798 | 2.999 | **0.000** |
| **HB T3** | **Nagelkerke R^2^** | **Exp(B)** | **pValue** |
| FNOS | 0.891 | 5.486 | **0.000** |
| **HB T4** | **Nagelkerke R^2^** | **Exp(B)** | **pValue** |
| FNOS | 0.938 | 13055934 | 0.993 |

| **HB T3** | **Nagelkerke R^2^** | **Case** | **FNOS** |
| --- | --- | --- | --- |
| Goodness of model | 0.908 | 75% | **95.8** |
| **HB T4** | **Nagelkerke R^2^** | **Case** | **FNOS** |
| Goodness of model | 0.938 | 76% | **97%** |

**Supplementary Table 5.** Multivariate analysis showing relationship between FNOS and HB outcome at different time (T1-T4). Binary logistic regression. Dependent variable: HB group; Independent variable: FNOS, assessed at T2, T3, T4. Precision indices of the regression model acquired with the categorical variable FNOS are also reported.
